# Medical education interventions influencing physician distribution into underserved communities: a scoping review

**DOI:** 10.1186/s12960-022-00726-z

**Published:** 2022-04-07

**Authors:** Asiana Elma, Muhammadhasan Nasser, Laurie Yang, Irene Chang, Dorothy Bakker, Lawrence Grierson

**Affiliations:** 1 Department of Family Medicine, Faculty of Health Sciences, David Braley Health Sciences Center, McMaster University, 100 Main St. W., Hamilton, ON L8P 1H6 Canada; 2 Bachelor of Health Sciences Program, Faculty of Health Sciences, McMaster University, Hamilton, Canada; 3 McMaster Community and Rural Education Program, McMaster University, Hamilton, Canada; 4 McMaster Education Research, Innovation and Theory, Faculty of Health Sciences, McMaster University, Hamilton, Canada

**Keywords:** Medical education, Undergraduate medical education, Graduate medical education, Selection criteria, Practice location, Health Workforce

## Abstract

**Background and objective:**

Physician maldistribution is a global problem that hinders patients’ abilities to access healthcare services. Medical education presents an opportunity to influence physicians towards meeting the healthcare needs of underserved communities when establishing their practice. Understanding the impact of educational interventions designed to offset physician maldistribution is crucial to informing health human resource strategies aimed at ensuring that the disposition of the physician workforce best serves the diverse needs of all patients and communities.

**Methods:**

A scoping review was conducted using a six-stage framework to help map current evidence on educational interventions designed to influence physicians’ decisions or intention to establish practice in underserved areas. A search strategy was developed and used to conduct database searches. Data were synthesized according to the types of interventions and the location in the medical education professional development trajectory, that influence physician intention or decision for rural and underserved practice locations.

**Results:**

There were 130 articles included in the review, categorized according to four categories: preferential admissions criteria, undergraduate training in underserved areas, postgraduate training in underserved areas, and financial incentives. A fifth category was constructed to reflect initiatives comprised of various combinations of these four interventions. Most studies demonstrated a positive impact on practice location, suggesting that selecting students from underserved or rural areas, requiring them to attend rural campuses, and/or participate in rural clerkships or rotations are influential in distributing physicians in underserved or rural locations. However, these studies may be confounded by various factors including rural origin, pre-existing interest in rural practice, and lifestyle. Articles also had various limitations including self-selection bias, and a lack of standard definition for underservedness.

**Conclusions:**

Various educational interventions can influence physician practice location: preferential admissions criteria, rural experiences during undergraduate and postgraduate medical training, and financial incentives. Educators and policymakers should consider the social identity, preferences, and motivations of aspiring physicians as they have considerable impact on the effectiveness of education initiatives designed to influence physician distribution in underserved locations.

**Supplementary Information:**

The online version contains supplementary material available at 10.1186/s12960-022-00726-z.

## Introduction

Inequitable distribution of physicians is a global problem [1, 2]. Half of the world’s population resides in rural areas but are served by less than a quarter of the physician workforce [1]. Consequently, rural-residing individuals have lower access to primary healthcare services [3–6], which contributes to higher incidence of chronic disease, injury, and mortality [7–9]. These disparities are even more pronounced amongst vulnerable and minority populations, including Indigenous and Francophone populations [10, 11]. Challenges of accessing primary care are also experienced in urban areas by individuals who are unhoused [12], recent immigrants [13–15] from certain ethnic or racial backgrounds [14, 16, 17], with low socioeconomic status [13–15, 18], individuals who are uninsured [19] and/or without full-time employment [14, 20].

There are few levers to encourage physicians to arrange practices in a way that offsets this maldistribution; however, medical education does present an opportunity. In the past, a variety of policy interventions have been implemented in response to the health disparities that are exacerbated by physician maldistribution. These include investments in ehealth and telemedicine to overcome communication and distance barriers in remote communities [5], increases in health human resources such as nurse practitioners and physician assistants, and the introduction of financial incentives to attract and retain physicians working in rural regions. However, evidence of the effectiveness of interventions such as these are limited [21, 22]. It is essential to find effective ways to address inequitable physician distribution, especially as the number of people challenged in accessing primary care continues to rise [23, 24].

There has been much discussion about the role health professions education can play in responding to healthcare and health system challenges. For instance, the World Health Organization (WHO) champions the importance of social accountability in medical schools, which it defines as “*the obligation to direct their education, research, and service activities towards addressing the priority health concerns of the community, region, and/or nation they have a mandate to serve. The priority health concerns are to be identified jointly by governments, health care organizations, health professionals and the public*” [25]. Accordingly, over the last two decades, the Canadian government has worked to expand medical school enrollment, assuming that graduating more physicians will improve overall access to care [26]. This has been accompanied by support for distributed medical education (DME) that accommodates the influx of new learners while also enhancing their exposure to authentic community-based learning environments in rural, remote, and other underserved areas [27]. Nevertheless, the challenge of access to primary care physicians persists.

Canadian medical education needs to expand its approach to influencing physician distribution and numerous interventions have been suggested [28]: the development of pipeline programs, enhanced admissions pathways, diversified learning contexts, and an increased emphasis on generalism throughout all stages of training [27, 29]. Many of these approaches have been tried and developing a strong understanding of those that are successful in influencing physician distribution is crucial. The objective of this scoping review is thus to understand the current literature pertaining to medical education initiatives designed to promote the uptake of family physicians in underserved areas. Through this review we intend to describe the education interventions that have been reported, their outcomes with respect to downstream physician practice in underserved areas, and any prevailing research gaps related to the relationship between education and physician distribution. This work differentiates from previous literature reviews, which were constrained to undergraduate training interventions [30, 31] or geographic regions [32, 33], inclusive of all types of primary care physicians [34], or relevant to the choice of family medicine specialty [35]. Specifically, this review adopts a global perspective considerate of interventions relevant to all stages of the medical training and maintains a specific focus on the distribution of family physicians.

## Methods

We employed Levac and colleagues’ [36] interpretation of Arksey and O’Malley’s scoping review framework [37] which is useful for covering a body of literature, identifying knowledge gaps, and informing future research or practice implications [38, 39]. The Preferred Reporting Items for Systematic Reviews and Meta-Analyses extension for Scoping Reviews (PRISMA-ScR) checklist guided translation of the results [40].

### Stage 1: defining the research objective

This scoping review describes medical education interventions implemented to promote family physician distribution in underserved rural, remote, or urban locations, and their outcomes.

### Stage 2: identifying relevant studies

When conducting scoping reviews, a balance needs to be struck between reviewing the vast and comprehensive literature that is available and the resources available to support the conduct of the study [36]. Accordingly, Inclusion and exclusion criteria were developed to ensure the scope of the search was appropriate for the research objective (Table 1).

**Table 1:** Inclusion and exclusion criteria

Inclusion:	Exclusion:
1. Participants are Family Physicians, with 'Family Medicine' as their core specialty in practice and can be inclusive of those with enhanced skill or focused practice 2. Participants that are completing undergraduate, postgraduate medical training and education, and/or working in Family practice 3. Studies that report on outcomes related to practice locations, practicing in underserved areas or intention to practice in underserved areas 4. Educational interventions in the context of the medical professional development trajectory (e.g., undergraduate, postgraduate medical education). Interventions can be inclusive of but not limited to preferential medical school admissions policies and selection criteria, undergraduate and postgraduate clinical placements that are described to influence the practice location decisions to underserved areas for participants 5. Studies written in the English language 6. Studies conducted in any country 7. All types of literature including case studies that employ all types of methodologies, such as qualitative, quantitative, mixed methods	1. Physicians from any other specialties or other allied healthcare professionals 2. Studies looking at outcomes relating to choosing medical specialty, or any other outcomes other than practice location, practicing in urban and/or rural areas, or intention to practice in underserved areas 3. Single papers that are published as commentaries, editorials, literature reviews, conference abstracts, doctoral theses 4. Studies in any other languages except English 5. Studies that include participants that are Primary Care Physicians but do not specify if it is inclusive of Family Physicians 6. Studies reporting on outcomes relating to perceptions, attitudes and/or preferences toward practicing in underserved settings

#### Types of participants and studies

Studies reporting on family physicians or “general practitioners” who a) provide longitudinal, continuous, and comprehensive care for patients experiencing common or long-term illnesses across all life stages and b) understand professional accountability to community health needs were included [41–44]. Studies on “primary care physicians”, comprising various specialties—including internal medicine, obstetrics and gynaecology, geriatrics, pediatrics, and family medicine—were excluded if they reported broadly on these practitioners without explicit mention of family physicians. We included all peer-reviewed articles that generated empirical evidence via any methodology (Table 1).

#### Underservedness of practice location

The review did not operationalize a standardized definition for underservedness. Given the global perspective, definitions of underservedness were expected to vary as a function of local contexts. Therefore, all definitions of underservedness were accepted.

#### Outcomes

Studies reporting on downstream practice locations and/or intentions to practice in underserved areas were included. Intention to practice in underserved areas was an outcome of interest as it is a proximal determinant of future behaviour [45].

Our search strategy was developed with support from an expert librarian. Database searches were conducted on Medline via Ovid, Web of Science, and Google Scholar. The strategy applied MeSH terms and keywords related to concepts of family physician, medical training, interventions, and practice location (Table 2). References were managed on Mendeley [46] and Covidence review management software was used for data extraction [47].

**Table 2:** Search strategy

Search Terms	1
“Physicians, Family” [MESH] OR “Physicians, Primary Care” [MESH] OR “General Practitioners” [MESH] OR “General Practitioners” [MESH] OR “General Practice” [MESH] OR “General practitioner*” [keyword] OR “Family practitioner*” [keyword] OR “Primary care practitioner*” [keyword] OR “Family physician*” [keyword] OR [Primary care physician*” [keyword] OR “family doctor*” [keyword] OR “Primary care doctor*” [keyword] OR “General practice physician*” [keyword] OR “general practice doctor” [keyword] AND “Education, Medical, Undergraduate” [MESH] OR “Education, Medical, Graduate” [MESH], “Residency training” [keyword] OR “Medical training” [keyword], OR “Clinical Clerkship” [MESH], OR “Family Medicine education” [keyword] OR “Preceptorship” [MESH] OR “Medical school admissions” [keyword] OR “School Admission Criteria” [MESH] AND “Professional Practice Location” [MESH] OR “practice location” [keyword] OR “rural practice*” [keyword] OR “urban practice*” [keyword]	2

### Stage 3: study selection

Each eligible study was screened via a two-stage process involving four reviewers (AE, MN, LY, IC). Reviewer discrepancies were resolved through regular team discussions.

### Stage 4: charting the data

A standard data extraction form was developed, piloted, and revised by the research team (Additional File 1). Extraction was completed by four team members (AE, MN, LY, IC).

### Stage 5: collate, summarize and report the results

Our analysis led to articulations of study characteristics, settings, definitions of underservedness, interventions, and main findings. We present frequency counts of study location and type characteristics. We also engaged in focused and open coding of the extracted data [48], developing general categories of education interventions according to their type, duration (where applicable), and location in the medical education professional development trajectory (e.g., undergraduate, postgraduate). We then constructed general definitions for each intervention category and summarized the associated findings pertaining to influencing practice or practice intentions in underserved areas.

### Stage 6: consultation exercise

We engaged our institution’s community and rural medical education leader (DB) as a co-author. As recommended by Levac and colleagues (2010), this individual offered an analytic consultation. This involved overview of our initial findings and feedback concerning the relevance and constraints of the reviewed literature with respect to known approaches to promoting an adequate geographic disposition of physicians. Subsequent analysis was then refined to reflect alignment with these insights.

## Results

**Fig. 1:**
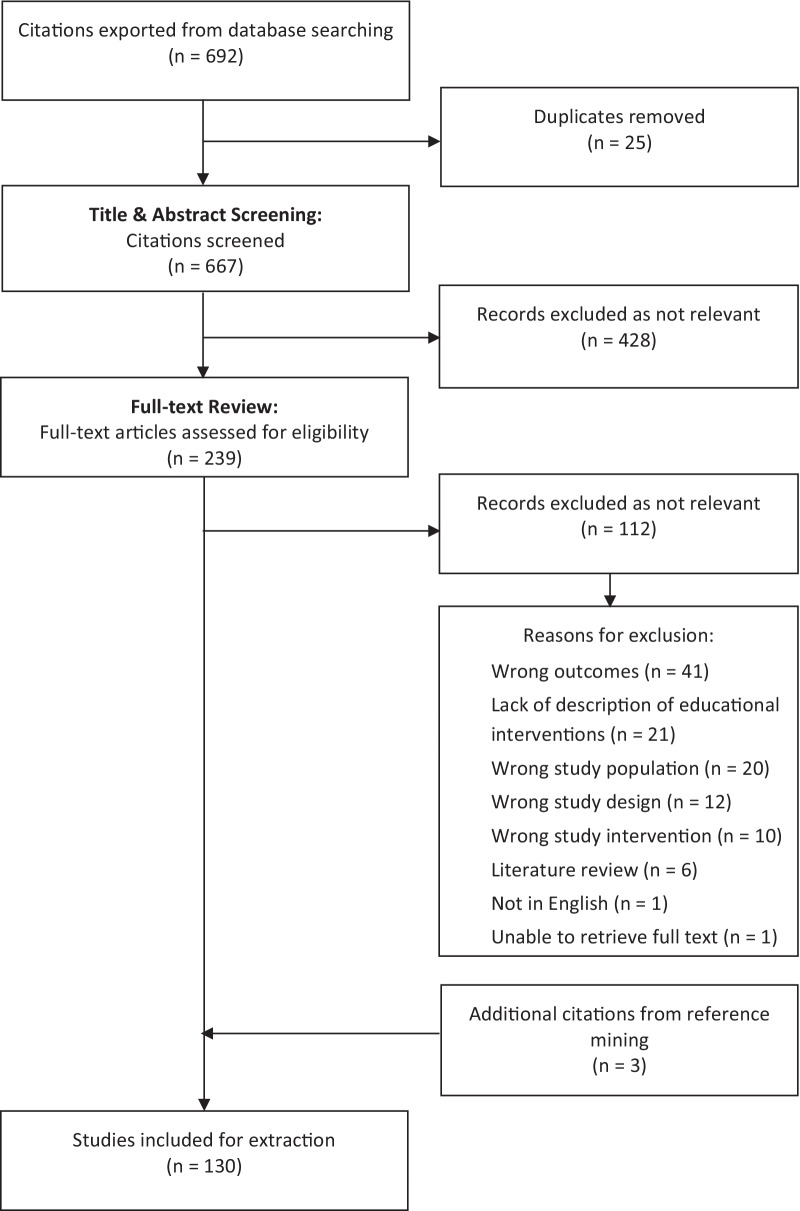
PRISMA flow chart depicting articles included and excluded throughout the screening stage

Database searching was completed in June 2021, identifying 692 potentially relevant articles. After duplicate removal, screening, and addition of new references, 130 eligible articles were included (Fig. 1).

### Study characteristics

The largest number of studies occurred in the United States, followed by Australia, Canada, and others (Table 3).

**Table 3:** Number and percentage of included studies according to study location

Study characteristics	Included studies (*n* = 130)
Study location	No. (%)
United States	75 (57.7)
Australia	22 (16.9)
Canada	22 (16.9)
Japan	3 (2.3)
New Zealand	2 (1.5)
Interregional*	2 (1.5)
Botswana	1 (0.8)
Germany	1 (0.8)
Ghana	1 (0.8)
South Africa	1 (0.8)

*Interregional: Studies that have been conducted across multiple countries. One (*n* = 1) was conducted in Australia and Canada and one (*n* = 1) study was conducted across five countries including Australia, South Africa, Sudan, Belgium, and Philippines

The vast majority of studies employed a cohort study design. Cross-sectional, mixed-methods, qualitative, and case–control designs were also employed (Table 4). Studies reporting on practice location outcomes relied primarily on single cohort or cross-sectional designs, which used administrative records or self-reported survey data to develop models of association between the educational intervention and practice outcomes. Mixed-methods studies relied on interview and self-reported survey data. Studies reporting on practice intentions predominantly used cross-sectional or qualitative designs, with few employing a cohort or mixed-methods design.

**Table 4:** Number and percentage of included studies according to study design

Study characteristics	Included studies (*n* = 130)
Study design	No. (%)
Cohort	56 (43.1)
Cross-sectional	52 (40.0)
Mixed methods	8 (6.2)
Qualitative	8 (6.2)
Case control	3 (2.3)
Other	3 (2.3)

#### Definitions of underservedness

Definitions of ‘*underservedness*’ varied considerably across all studies. In some cases, it was defined in terms of the proportion of residents from various ethnic or cultural backgrounds [49–55], with low-socioeconomic status [49, 51, 52, 56–58], who live in poverty [54, 58–61], or who are of older age [52]. Australian studies particularly used the Index of Relative Socioeconomic Advantage and Disadvantage classification system to capture the economic and social conditions of people residing in a particular area [62, 63]. American studies also employed the constructs of the Health Professions Shortage Area (HPSA; defined as an area with less than 1 primary care physician per 3,500 population) [52, 54, 58, 59, 64–72], and the medically underserved area (MUA; defined as areas, where 40% of patients receive Medicaid or are uninsured) [52, 58, 64, 70–73].

Although the review was inclusive of educational interventions designed to promote practice in any type of underserved community, the vast majority of studies reported on outcomes pertaining to practice in rural areas, which were defined in numerous ways. Several Australian studies used the Australian Standard Geographical Classification [55, 62, 63, 71, 74–79], the Rural Remote Metropolitan Area (RRMA) [80–85] or used the Modified Monash Model (MMM) [86–88]. Other studies used population metrics or distances from metropolitan areas to define rural areas. For example, Rolfe and colleagues (1995) defined major metropolitan areas as having populations greater than 100,000 people and remote areas by their distance from metropolitan areas [89]. Canadian studies also used various definitions. Rourke (2018) and Mathews (2017) used Statistics Canada’s population-based definition of rural areas [53, 90], while Barrett and colleagues defined rurality according to both population size and proximity to an urban center [32]. Studies from the United States used Rural–Urban Continuum Area Codes (RUCAC) [69, 70, 91–101], non-metropolitan Statistical Areas [102–108], and the number of individuals who lack access to care due to cultural and economic factors [54] to index rurality. Japanese studies defined rurality in terms of municipalities with five or less physicians, municipalities with a 5–100 000 physician-to-population ratio, or municipalities with population under 20,000 and a less than 100–100 000 physician-to-population ratio [63, 109].

In a small number of studies, the concepts of rurality and underservedness were treated as separate entities [70, 72, 110].

### Types of interventions

Educational interventions described as influencing practice location or intention to practice in underserved areas aligned with four categories: preferential admissions criteria, undergraduate training in underserved areas, postgraduate training in underserved areas, and financial incentives. A fifth category was constructed to reflect initiatives comprised of various combinations of these four interventions (Table 5).

**Table 5:** Number and percentage of the different types of medical education interventions

Education interventions	Included studies (*n* = 130) No. (%)
Singular interventions	86/130 (66.2)
Preferential admissions	3 (3.5)
Rural undergraduate training	37 (43.0)
Rural postgraduate training	42 (48.8)
Financial incentives	4 (4.7)
Multiple interventions	44/130 (33.8)
Admissions and rural undergraduate training	11 (25.0)
Rural undergraduate and postgraduate training	11 (25.0)
Admissions, rural undergraduate training, and financial incentives	9 (20.5)
Admissions, rural undergraduate, and postgraduate training	6 (13.6)
Rural postgraduate training and financial incentives	3 (6.8)
Admissions, rural undergraduate and postgraduate training and financial incentives	2 (4.5)
Rural postgraduate training and financial incentives	1 (2.3)
Rural undergraduate and postgraduate training and financial incentives	1 (2.3)

#### Preferential admissions criteria

Three studies investigated the independent influence of medical school admissions policies that contemplate the selection of applicants with certain socio-cultural backgrounds and/or who are from targeted underserved areas on eventual practice location [62, 111] or intention to practice in underserved areas [49]. A WHO study revealed that aspiring physicians selected via admissions policy that favoured those with a rural small-town background and/or who expressed specific desire to practice rural family medicine were significantly more likely to practice rural family practice than those not selected under this policy (RR 3.9, CI 2.7–5.7, *P* < 0.001) [111]. Similarly, an Australian study revealed students selected on the basis of Indigenous identity, rural upbringing, or socioeconomic disadvantage demonstrated a twofold increase in the odds of practicing in a socioeconomically disadvantaged community [62]. An interregional study reported that students selected from Indigenous, African, or rural populations reported greater intention to practice with underserved populations in rural or remote areas after graduation (*p* = 0.000) [49].

#### Undergraduate training experiences in underserved areas

Thirty-six studies reported on the relationship between undergraduate training in underserved areas and eventual practice location [50, 51, 56, 57, 63, 71, 74, 75, 80, 86, 87, 91, 92, 110, 112–124] or intentions to practice in underserved areas [52, 81, 93, 123–130]. This included training at medical education institutions in an underserved location (usually rural) [91, 128], shorter opportunities for medical students to participate in clerkships, internships, externships, or placements in any such practice setting (e.g., hospital, family practice) for any duration [50–52, 56, 57, 63, 71, 75, 80, 81, 86, 87, 92, 110, 112–116, 118–121, 125, 127, 129, 130], and combinations of clinical placements with specific non-clinical curricula [50, 57, 113, 122].

Most studies in this category demonstrated a positive effect on practice outcomes in underserved areas [50, 51, 56, 57, 63, 71, 75, 86, 91, 92, 110, 112–117, 119, 120, 130], although two studies reported equivocal findings [74, 117]. For example, an Australian cohort study reported that graduates who spent at least 1 year at a rural clinical school were significantly more likely to practice in rural areas than those who did not (27 vs. 7%) [75]. Notably, the duration of rural undergraduate training varied across studies, ranging from 6 weeks to more than 1 year, with some reporting that associations with eventual practice in underserved locations were stronger the longer the duration of the placement [86, 87].

The majority of studies reporting on the effect of rural undergraduate training on intentions to practice described positive outcomes [53, 81, 122, 123, 127–130]. An American cross-sectional study indicated that students placed in underserved locations had greater odds of reporting intention to work in such communities at graduation (OR 9.40, 95% CI 4.66–19.96) [52]. However, one Canadian study reported low impact of these types of interventions [125] and others reported equivocal impact on practice location intentions [93, 123, 124, 126].

#### Postgraduate training experience in underserved areas

Forty-two studies reported on the relationship between postgraduate training in underserved areas and eventual practice location [41–77, 82, 89, 90, 94, 95, 102, 131–138, 140–154], practice intentions [67, 83, 155, 156] or both [157]. This training included enrollment in postgraduate training at a rural campus location [58, 64, 69, 76, 90, 94, 95, 131, 132, 134–136, 138, 139, 144–146, 149, 151, 154, 155] and opportunities for residents to participate in rotations, internships, externships, or placements in any practice setting (e.g., hospital, family practice) in an underserved area for short-term (2–3 weeks), intermediate (4 weeks to 1 year) and long-term (≥ 1 year) durations [58, 59, 65, 66, 77, 82, 83, 89, 102, 133, 137, 140, 141, 146–148, 150, 152–154, 156, 157].

Largely, these studies indicated a positive association between completing residency or postgraduate training experiences in underserved areas and eventual practice in underserved locations [58, 65, 66, 77, 82, 89, 90, 131–133, 136, 137, 140–150, 152–154, 157]. Notably, several studies found that graduates of rural residency programs practice in close proximity to where they completed their postgraduate training [90, 131, 132, 136, 144–146, 149, 151]. For example, a cohort study of family practice residency programs in various regions in the United States indicated that most graduates (76.8%) practice within 100-mile radius of their residency program [151]. Another American study reported that this relationship is greater for more recent cohorts relative to earlier cohorts [136]. Two studies demonstrated equivocal findings [59, 102]. One Canadian qualitative study reported a potential negative relationship, where graduates who participated in rural family medicine describe practicing in urban areas with no intention to move to a rural practice location, despite positive experiences with their training [139].

Five studies described the impact of rural postgraduate training experiences on practice location intentions [67, 83, 155–157], reporting equivocal findings. One study reported that graduates who completed rural rotations during residency expressed greater intentions to practice in rural areas [157], while another study reported that they did not [82]. One study reported that a significant association between rural education and rural practice intentions was more likely for senior than junior residents [155].

#### Financial incentives

Four studies reported on educationally relevant financial incentives provided during medical training and designed to promote practice in underserved areas [60, 96, 158, 159]. Specifically, three studies reported on tuition and living expenses provided to medical learners via the United States’ National Health Services Corps (NHSC), which conveys in exchange for 2 years of service in an underserved community [96, 158, 159]. These studies report that the program is influential in encouraging physicians to work in underserved communities; however, the participants’ commitment to serving the assigned communities beyond the formal obligation varied. When these physicians began practicing at their assigned practice, 14% of the NHSC physicians anticipated remaining in that location longer than 5 years, whereas 70% of the non-NHSC physicians intended to continue practicing in underserved areas for longer than 5 years (OR 0.07, P < 0.001) [159]. One study reported that most learners left their assigned practices within months of concluding their obligation [96].

### Combinations of interventions

Forty-four studies reported on the influence of two or more of the above-described interventions on eventual practice location or intended practice locations [53–55, 61, 68–70, 72, 73, 78, 79, 82, 83, 88, 96–100, 102–108, 160–177]; See Table 4]. Several of these studies reported that a combination of preferential admissions criteria and opportunities for rural training experience at both the undergraduate and postgraduate levels is influential in physicians choosing rural practice [53–55, 85, 173, 174]. Two studies posited that the specific combination of selecting students from rural areas and providing opportunities for clinical training in rural areas during the third year of medical school was the most influential in promoting eventual rural practice [69, 84]; however, these results are potentially confounded insofar that the students involved may have had a pre-existing interest in rural practice.

## Discussion

This review mapped the literature reporting on educational interventions designed to influence family physicians to practice in underserved areas. The review highlights that many training institutions around the world have made such efforts—with a particular focus on increasing the uptake of practitioners in rural areas. Summarily, the literature outlines preferential admissions policies, placements in relevant practice settings during undergraduate and postgraduate training, financial support in exchange for time-limited-service agreements, and various combinations of these approaches as relevant. Overall, the majority of studies report positive outcomes associated with these interventions.

The review highlights that allocating medical school seats to those from or predisposed to practice in underserved areas may be an effective approach to promoting practice in these areas; but also, that this may not be entirely sufficient. Specialized non-clinical curriculum focusing on rural-residing or traditionally underserved patients [50, 92], workshops and seminars [57], training at a rural medical school [86, 87], and rural experiences provided through short-term and long-term placements, were all also influential in promoting practice in rural and underserved areas. Through these interventions, students may develop positive perceptions about practice in underserved communities, develop the appropriate skills to do so [137], and receive important mentorship from those who have expertise in these communities [57, 119]. The review suggested that combinations of admissions, undergraduate and postgraduate placement, and financial incentives may be particularly effective; however, did not indicate which combination of interventions is most effective in graduating physicians into underserved areas. It is important to highlight that learning experiences may also discourage students from practice in underserved areas. For instance, they may develop perceptions that the work and lifestyle are overly challenging [50, 57, 74]. Personal reasons, such as those related to family planning and spousal preferences, may also push learners away from these practice locations [126]. Given this, medical schools should consider the interaction between educational and personal factors when developing experiences for learners. In this regard, the simple introduction of interventions can be thought of as having a potential positive effect on the *hidden curriculum* of medical education [178]. When experiences in rural and underserved communities are prioritized within admissions and teaching activities, supported by knowledgeable mentors, and encouraged with funding, this type of practice may be perceived as more valuable.

The review also elucidates how evaluations of these educational interventions are largely situated within the medical education context and do not consider how they interact with healthcare initiatives or policies that operate outside of the training environment. For instance, numerous underserved communities mount their own projects to influence physician recruitment and retention, including monetary and lifestyle incentives, offsetting overhead costs, housing support, and fundraising activities for recruitment campaigns [179–183]. Future research may consider how these grassroots programs interact with educational interventions to promote the uptake of family physicians in underserved communities. Similarly, many medical schools now have admissions policies that contemplate applicant selection with respect to their equity, diversity, and inclusion commitments, with minimal focus on resolving the physician maldistribution challenge. In Canada, some examples include admissions pathways for Black [183–187] and Indigenous [189–195] applicants. With respect to the evidence demonstrating a relationship between physician social identity characteristics and eventual practice location or practice intentions [196–200], there may be an unintended downstream relationship between these admissions processes and the practice intentions or locations of the matriculants. In this regard, we encourage evaluations of these policies to extend beyond the diversity of resulting medical school classes so as to also formally consider the eventual impacts on physician distribution. Considerations for medical schools to design and adopt mission statements that reflect the social responsibility of graduating physicians into underserved communities present another potential avenue for influencing the health workforce outcomes as medical schools’ social mission content was reported to be a significant predictor of physician output in medically underserved areas and populations [201]. However, it is unclear if this effect was a result of the institution’s orientation or if medical learners were predisposed to work in the underserved areas and subsequently self-selected into institutions that align with their practice intentions. The review also revealed that a vast majority of the studies have a singular focus on educational interventions situated to influence physician disposition in rural or remote areas, with less consideration for underserved communities in urban areas. Future program evaluations should consider designing curricula and medical education initiatives that expose learners to working in underserved urban communities as populations with certain ethnic, cultural and/or socioeconomic backgrounds residing in urban locations experience challenges with accessing primary care [12–18, 20].

The review has some notable limitations. Included studies were heterogeneous with respect to designs, interventions, and definitions of underservedness. Accordingly, our findings were summarized on a broader level, which inherently suppresses some of the unique features of different approaches. Second, numerous studies were single cohort or cross-sectional in design and many used self-reported survey data. We recommend researchers in this area conduct more longitudinal studies [202]. This would strengthen the overall quality of the evidence. Furthermore, many studies did not account for student background or pre-existing interest in practicing in underserved areas, making it challenging to understand the true, independent impact of interventions. Finally, our review may also be beset by considerable publication bias. It is likely that the strong representation of positive findings emanates from a tendency for medical education scholars to only seek publication of evaluations that reveal positive outcomes vis-à-vis programmatic objectives. In this case, instances where educational interventions were not successful may not be captured within this review.

## Conclusions

Medical education may play an important role in addressing the challenges underserved communities face in accessing primary care family physicians. Various educational interventions can influence physician practice location: preferential admissions criteria, rural experiences during undergraduate and postgraduate medical training, and financial incentives. Effective strategies must also consider the social identity, preferences, and motivations of aspiring physicians as they have considerable impact on the effectiveness of education initiatives designed to promote practice in underserved settings.

## Supplementary Information

**Additional file 1.** Data extraction template.

## Data Availability

Data sharing is not applicable to this article as no data sets were generated or analysed during the current study.
