# Supplementary material for: Medical education interventions influencing physician distribution into underserved communities: a scoping review
Source: Hum Resour Health. 2022 Apr 7;20:31. doi: 10.1186/s12960-022-00726-z (PMC8991572; doi:10.1186/s12960-022-00726-z)
Supplement: Supplementary file 1 — Additional file 1. Data extraction template. [file 12960_2022_726_MOESM1_ESM.docx]

**Appendix 1.** Data extraction template

| **General Information** | |
| --- | --- |
| Study Details | Title of paper; author’s name; year study was conducted |
| Country | Country in which the study was conducted |
| **Characteristics of Included Studies** | |
| Aim or Objective of Study | As described by the authors |
| Study Design | The way the study was conducted |
| Start and End Date of Study | When the study was conducted; the time period of the datasets used in the study (e.g., dataset of graduating medical students from 1999 to 2010). |
| Participants | Total number of participants; participant characteristics (e.g., gender, age, role) |
| Recruitment of Participants | The way participants were recruited (where applicable) |
| **Intervention** | |
| Type of Educational Intervention | The nature of the intervention |
| Description of Educational intervention | A description of the intervention including details on the location of the intervention (e.g., institution name, urban/rural description), the duration of the intervention, the type of exposures that are part of the intervention. |
| **Outcomes** | |
| Outcomes Measured | The main outcomes that were measured in the study |
| Urban/Rural Definition | As described by the authors |
| Method of Outcome Measurement | The way the outcomes were measured in the study |
| **Findings** |  |
| Study’s Main Findings | Describes the outcomes of the educational interventions, main points in the discussion or conclusions described by the author |
| Strengths & Limitations | The limitations of the study’s described by the authors |
| Recommendations and/or Future Directions | Description of recommendations for educational interventions to enhance physician distribution (as described in the article) |
